# Cognitive trajectories in older adults and associated mortality and predictors

**DOI:** 10.1007/s00127-025-02862-y

**Published:** 2025-03-06

**Authors:** Elena Lobo, Concepción De la Cámara, Patricia Gracia-García, Pedro Saz, Raúl López-Antón, Antonio Lobo

**Affiliations:** 1 Department of Preventive Medicine and Public Health, Universidad de Zaragoza, Zaragoza, 50009 Spain; 2 Instituto de Investigación Sanitaria Aragón (IIS Aragón), Zaragoza, 50009 Spain; 3 Centro de Investigación Biomédica en Red de Salud Mental (CIBERSAM), Ministry of Science and Innovation, Madrid, 28029 Spain; 4 Department of Medicine and Psychiatry, Universidad de Zaragoza, Zaragoza, 50009 Spain; 5 Psychiatry Service, Hospital Clínico Universitario, Zaragoza, 50009 Spain; 6 Psychiatry Service, Hospital Universitario Miguel Servet, Zaragoza, 50009 Spain; 7 Department of Sociology and Psychology, Universidad de Zaragoza, Zaragoza, 50001 Spain

**Keywords:** Cognitive path, Death risk, Outcome, Community sample

## Abstract

**Purpose:**

To test the hypotheses that declining cognitive aging trajectories would increase mortality risk and that predictors of mortality would differ between trajectory groups.

**Methods:**

This is a sub-study of the ZARADEMP project studying dementia and depression in older adults aged 55 years or more, conducted in Zaragoza, Spain, including 2403 cognitively healthy individuals who had completed at least three of the four waves in a 12-year follow-up. The three cognitive trajectories previously identified were based on the Mini-Mental State Examination (MMSE). Mortality information obtained from the city’s official population registry was registered up to 6 years after the end of the fourth wave of the study. Cox proportional hazard regression analyses for analyzing the risk of death were performed globally and for each cognitive trajectory.

**Results:**

At follow-up, 42.4% of the participants had died. Individuals in class 2-moderate-stable and in class 3-low-and-declining had a 24% and 96%, respectively, higher risk of mortality than those in class 1-high-to-moderate. Those younger and women showed significant lower risks of death in all the classes. Being single, with diabetes, dependency in basic Activities of Daily Living, ex-drinkers, smokers, and ex-smokers increased the risk in class 2. Hypertension showed a higher risk of death in the high-to-moderate group. In the low-and-declining trajectory, anxiety nearly tripled the risk of death.

**Conclusion:**

Trajectories with cognitive decline are associated with higher mortality, with the risk of death showing a gradient. Predictors of mortality differ by cognitive trajectory; the differences being observed even among the cognitively healthier groups.

## Introduction

The absence of cognitive impairment has been considered one of the main components of healthy aging [1]. On the contrary, cognitive decline has been associated with negative outcomes, including higher mortality [2, 3].

Cognitive impairment has been linked with various systemic conditions associated with a higher risk of death, such as cardiovascular diseases and diabetes, which share common pathophysiological mechanisms like inflammation and vascular dysfunction [4]. Moreover, neurodegenerative diseases with cognitive impairment like Alzheimer’s disease and other types of dementia, but also mild cognitive impairment /MCI) had higher mortality rates than those with normal cognition (hazard risks: 2.61–5.20) [5].

Behavioral and social factors associated with cognitive decline may also partially explain the mortality risk. Cognitive decline influences an individual’s ability to perform activities of daily living (ADLs), manage health-related behaviors, and adhere to medical treatments. Impairments in cognitive domains can lead to poor disease management, medication non-adherence, unhealthy lifestyle choices, and dependency, thereby exacerbating negative health outcomes and higher rates of institutionalization, both of which are associated with increased mortality [6–8]. Also, cognitive deterioration often leads to social isolation and depression [9], both of which are independently associated with poorer health outcomes and increased mortality [10, 11].

Particular interest in cognitive function and health outcomes may have the study of cognitive trajectories. The study of cognitive paths through aging has emerged as a more comprehensive perspective for understanding the natural process of cognition while we age [12]. This approach emphasizes the benefits of capturing the complex and dynamic nature of cognitive processes and highlights the variability in cognitive trajectories among individuals [13]. Using longitudinal data offers significant advantages over cross-sectional measures by identifying latent classes of cognitive changes over time [14]. Previous reports have documented that individuals group in two to seven distinct cognitive trajectories through aging [12], and, interestingly, that predictors of class membership are different among the groups, suggesting that protective factors might be different and not just the opposite of risk factors [12, 14].

Some studies have associated cognitive trajectories with health outcomes. Individuals classified in trajectories of rapid decline might have an increased risk of incident dementia, frailty, and dependency in activities of daily living (ADLs) [15–18]. While poor health outcomes might be related to mortality, few studies have specifically measured the impact of being grouped in a cognitive trajectory with the risk of death. Those studies come mainly from American or Asian samples, with older adults of 65 or more years [15, 16, 19–21], and there is no data from the South of Europe nor studies in younger populations that might provide relevant information for earlier interventions. Moreover, predictors of mortality in different trajectory groups, which were not explored in these previous studies could also provide relevant information for preventive strategies.

The present paper aimed to test the hypothesis that trajectories with decline would be associated with higher mortality risk in a sample of adults aged 55 years or more. We also hypothesize that predictors of mortality differ between trajectory groups.

## Methods

This is a sub-study of the ZARADEMP project, a longitudinal population-based study of dementia and depression in older adults of 55 years or more, conducted in Zaragoza, Spain. A random, representative sample, stratified with proportional allocation by age and sex, including institutionalized individuals was recruited and followed for twelve years in four waves. A detailed explanation of the general methodology can be found elsewhere [22].

The study included 4,803 participants, with an overall participation rate of 79.5%. A two-phase case finding for dementia in each wave was used. Dementia and “subsyndromal” dementia cases at baseline, identified with the Geriatric Mental State (GMS), with its cognitive section and its Automated Geriatric Examination for Computer Assisted Taxonomy package (AGECAT) criteria [23], were excluded in the follow-up.

### Assessment of cognitive function

Cognitive functions such as orientation in time and space, memory, attention, calculation, language, and visuoperception, were assessed with validated, Spanish versions of the following international instruments: cognitive section of the GMS and AGECAT, and the Mini-Mental State Examination, MMSE [24, 25].

In phase 1, in each wave, lay interviewers specially trained (senior medical students) administered the instruments in the elderly’s home and obtained cognitive data, thoroughly reviewed by the research psychiatrists supervising the interviewers individually. In phase 2, the research psychiatrists re-examined, two months later, and blind to the results of phase 1, the probable cases of dementia. For the present analysis, only the performance in the MMSE in phase 1 was used.

Trajectories of change over time in cognitive function had been modeled using growth mixture models (GMM) and published in a previous manuscript [26]. Including data from participants with at least three measurements of MMSE, with different baseline ages and number of time points, this technique resulted in individuals being classified into clusters with similar trajectories according to their longitudinal data of MMSE at each wave, assuming that individual differences in trajectories can be summarized by a finite set of different polynomial functions for age or time [27, 28]. The best fitting age-adjusted model was a 3-class growth model, comprising classes 1-high-to-moderate, 2-moderate-stable, and, 3-low-and-declining cognitive function over time, with 21.2%, 65.7 and 9.9% of the sample, respectively [26].

### Other variables

At the baseline visit, the interview included information on age, sex, marital status, education, and health behaviors such as alcohol and smoking. Education was categorized as Illiterate, Primary (Elementary School, complete or incomplete) and High (High School and/or University). The History and Aetiology Schedule (HAS) was used and functional dependency was evaluated by disability scales (Katz’s Index) for basic activities of daily living (bADLs), and Lawton and Brody scale for instrumental ADLs (iADLs). The European Studies of Dementia (EURODEM) Risk Factors Questionnaire was used to gather data on diabetes and hypertension [29]. The GMS interview and the respective sections in the AGECAT computer system provided information on depression and anxiety syndromes. After symptom assessment (AGECAT Stage 1), a diagnosis of depression emerges from Stage II. In this stage, a computer program compares syndrome clusters (e.g., dementia, depression, anxiety) to reach a final diagnosis, recorded as either a diagnostic “subsyndromal” (confidence levels 1 and 2) or a diagnostic “case” (confidence levels 3). The “cases” level was used in this particular study.

### Ascertainment of mortality

The methods for determining deaths in the ZARADEMP projects have been previously published [2]. The official population registry in the city was the reliable source for the identification of all-cause mortality of each participant. This information was completed and verified via death certificate, which included accurate information on the day, month, and year of death. Days from birth to the date of death were calculated for each subject, and those individuals remaining alive or missing (emigrated, not localizable) six years after the fourth wave were included in the analysis as censured.

### Data analysis

Mean and standard deviation (SD) for continuous variables and proportions for categorical variables were used to describe baseline characteristics. To compare differences, the Chi-squared test and T-Student test were used. Cox proportional hazards regression models were used to analyze associations between cognitive trajectories (Classes 1 to 3) and time to death during follow-up, as well as to analyze the predictors of mortality within each trajectory. Hazard Ratios (HR) with 95% confidence intervals (CI) were estimated. Subjects not registered as dead were considered censored at the last date when follow-up information was assessed, or lost to follow-up, whichever occurred first. Sociodemographic, medical, and lifestyle variables were included as potential confounders. All analyses were implemented using SPSS software v.26 [30].

## Results

The final sample was composed of 2403 subjects, and the baseline characteristics have been described before [26]. In summary, they were more often women and their mean age was 70.3 years old. The majority of them had a low level of education. The frequency of hypertension and diabetes was 67% and 11.7%, respectively. Depression and anxiety syndromes were present in 15.8% and 4% of the subjects. The majority (60.5%) never drank, and most of them were non-smokers (64.3%).

At follow-up, after 18 years from baseline, 42.4% of the participants had died. As shown in Table 1, at baseline and compared with those alive/censored, the individuals who died were older and more frequently men, illiterate, single or widowed, with hypertension, diabetes, and dependency on ADL; habitual drinkers of alcohol, smokers and ex-drinkers and ex-smokers. The MMSE score at baseline was lower than in those alive/censored.

**Table 1:** Baseline characteristics of participants, by survival status (*N* = 2403)

	Death cases^b^ *n* (%)/ mean (SD)	Survivors/censored^c^ *n* (%) / mean (SD)	*p*-value
**Women**	494 (48.4)	848 (61.3)	0.000
**Age**	74.7 (8.2)	67 (6)	0.000
**Education**			
**Illiterate**	87 (8.6)	64 (4.6)	
**Primary**	744 (73.4)	1029 (74.7)	0.000
**High**	182 (18)	284 (20.6)	
**Marital status**			0.000
Couple	600 (58.8)	1029 (74.4)	
Widowed	297 (29.1)	226 (16.3)	
Single^a^	123 (12.1)	128 (9.3)	
**Hypertension**	744 (73.1)	866 (62.7)	0.000
**Diabetes**	137 (13.5)	145 (10.6)	0.026
**Depression**	174 (17.8)	205 (15.2)	0.096
**Anxiety**	47 (4.6)	48 (3.5)	0.157
**iADLs Dependency**	129 (12.7)	53 (3.8)	0.000
**bADLs Dependency**	66 (6.5)	36 (2.6)	0.000
**Alocohol**			
Never	578 (56.7)	875 (63.4)	0.000
Ocassional	39 (3.8)	77 (5.6)	
Habitual	257 (25.2)	320 (23.2)	
Ex-drinker	146 (14.3)	109 (7.9)	
**Smoking status**			0.000
Non-smoker	600 (58.8)	947 (68.5)	
Smoker and Ex-smoker	420 (41.2)	436 (31.5)	
**MMSE at baseline**	27.1 (2.4)	27.9 (2)	0.000
**Cognitive Trajectories**			
Class 1-high-to-moderate	190 (18.6)	320 (23.1)	
Class 2- moderate-stable	703 (68.9)	953 (68.9)	0.000
Class 3- low-and-declining	127 (12.5)	110 (8)	

*Note. iADLS: instrumental Activities of Daily Living. bADLs: basic Activities of Daily Living. MMSE: Mini-Mental Status Examination.*^a^*Single*,* separated.*^b^*N* = *1020.*^c^*N* = *1383*

Referring to the cognitive trajectories, compared with survivors/censored, the individuals who died had been more frequently included in class 3-low-and-declining cognitive function over time (12.5% vs. 8%), and less frequently in class 1-high-to-moderate (18.6 vs. 23.1), while no differences were observed in those included in class 2-moderate-stable (68.9) (Table 1).

In the Cox proportional hazards regression analyses, after controlling for potential confounders, cognitive trajectories were associated with the risk of death. Compared with those in class-1-high-to-moderate, those grouped in class 2-moderate-stable and in class 3-low-and-declining had a 24% (HR = 1.24, 95% CI (1.03–1.48), *p* = 0.021) and 96% (HR = 1.96, 95% CI (1.44–2.66), *p* = 0.000), respectively, higher risk of mortality (Table 2). When comparing classes 2 and 3, those in class 3-low-and-declining had a 57% higher risk of death (HR = 1.57, 95% CI (1.25–1.98) than those in class 2-moderate-stable (not in tables). Referring covariates, sex, age, civil status, hypertension, diabetes, anxiety, dependency in basic and instrumental ADLs, alcohol exdrinkers and tobacco smokers/exsmokers were associated with the risk of death.

**Table 2:** Hazard ratios (95% CI) for the association between cognitive trajectories and risk of mortality (crude and adjusted)

	Crude			Adjusted		
	HR	IC95%		HR	IC 95%	
**Cognitive trajectories**						
Class 2-moderate-stable	1.25*	1.06	1.46	1.24*	1.03	1.49
Class 3-low-and-declining	1.71**	1.37	2.15	1.96**	1.44	2.66
**Femenine sex**	0.69**	0.61	0.78	0.59**	0.48	0.72
**Age**	1.12**	1.11	1.13	1.12**	1.11	1.13
**Education (ref. High)**						
Illiterate	1.79**	1.38	2.31	1.13	0.84	1.51
Primary	1.12	0.95	1.31	0.93	0.78	1.11
**Civil status (ref. couple)**						
Widowed	1.9**	1.65	2.19	1.20*	1.01	1.42
Single ^a^	1.47**	1.21	1.79	1. 38*	1.11	1.71
**MMSE at baseline**	0.90**	0.88	0.92	0.99	0.96	1.03
**Hypertension**	1.48**	1.29	1.70	1.19*	1.03	1.38
**Diabetes**	1.23*	1.03	1.47	1.24*	1.02	1.50
**Depression**	1.16	0.99	1.37	1.12	0.94	1.34
**Anxiety**	1.30	0.97	1.74	1.64*	1.20	2.23
**iADLs Dependency**	2.81**	2.33	3.38	1.25*	1.01	1.55
**bADLs Dependency**	2.33**	1.81	2.99	1.52*	1.15	2.0.1
**Alcohol (ref. never)**						
Occasional	0.81	0.59	1.27	0.77	0.55	1.08
Habitual	1.14	0.99	1.33	0.98	0.83	1.17
Exdrinker	1.70**	1.41	2.03	1.26*	1.03	1.55
**Smoker/Ex-smoker**	1.35**	1.20	1.53	1.40**	1.17	1.67

*Note. iADLS: instrumental Activities of Daily Living. bADLs: basic Activities of Daily Living. MMSE: Mini-Mental Status Examination.*^a^*Single*,* separated.* *p < 0.05. **p < *0.001*

The class-specific hazard ratios (HRs) for survival, in Table 3, showed in all the groups a protection effect for feminine sex and a 12% (or above) increased mortality risk for those older. Subjects with hypertension showed a higher risk of death in the high-to-moderate group (class 1, HR: 1.43, 95%CI (1.01, 2.03)). In the moderate-stable class 2, a higher risk of mortality was observed for single individuals (HR: 1.35, 95%CI (1.04, 1.74)) compared to being with a couple; and for subjects with diabetes (HR:1.28, 95%CI (1.01, 1.60)), dependency in basic ADLs (HR: 1.62, 95%CI (1.16, 2.27) respectively), exdrinkers (HR: 1.35, 95%CI (1.06, 1.73)), and smokers and ex-smokers (HR: 1.38, 95%CI (1.11, 1.72)). Anxiety increased the risk of mortality for those in the low-and-declining class 3, and the magnitude of the association was higher than for age or sex (HR: 2.88, 95% CI (1.45, 5.75)).

**Table 3:** Class-specific risk factors for mortality in each trajectory group

	Class 1- High-to-moderate		Class 2-moderate-stable		Class 3-low-and-declining	
	HR	95% CI	HR	95% CI	HR	95% CI
**Femenine sex**	0.61*	0.40, 0.95	0.60**	0.47, 0.77	0.48*	0.25, 0.91
**Age**	1.13**	1.10, 1.16	1.12**	1.10, 1.13	1.13**	1.09, 1.17
**Civil status (Ref. Coupled)**						
Widowed	1.23	0.81, 1.88	1.21	0.98, 1.48	0.93	0.56, 1.53
Single ^a^	1.41	0.87, 2.29	1.35*	1.04, 1.74	1.25	0.58, 2.68
**Education level (ref. Higher)**						
Illiterate	0.58	0.08, 4.35	1.10	0.77, 1.58	1.52	0.5, 4.59
Primary	0.97	0.71, 1.32	0.87	0.69, 1.09	1.22	0.43, 3.45
**MMSE at baseline**	1.14	0.89, 1.46	0.97	0.93, 1.02	1.04	0.98, 1.11
**Hypertension**	1.43*	1.01, 2.03	1,16	0.97, 1.38	0.86	0.53, 1.39
**Diabetes**	1.28	0.80, 2.03	1.28*	1.01, 1.60	0.93	0.52, 1.67
**Depression**	1.32	0.81, 2.17	1.09	0.88, 1.36	0.99	0.62, 1.60
**Anxiety**	1.76	0.79, 3.90	1.39	0.93, 2.07	2.88*	1.45, 5.75
**ADLs instrumental**	1.21	0.64, 2.27	1.29	0.99, 1.68	1.36	0.79, 2.34
**ADLs basic**	1.19	0.57, 2.51	1.62*	1.16, 2.27	1.37	0.62, 3.06
**Alcohol (Ref Never)**						
Occasional	0.70	0.35, 1.44	0.81	0.55, 1.21	0.42	0.05, 3.17
Habitual	1.04	0.72, 1.49	0.99	0.81, 1.23	0.76	0.41, 1.43
Exdrinker	1.07	0.65, 1.76	1.35*	1.06, 1.73	1.03	0.51, 2.09
**Smoker and exsmoker (Ref. non smoker)**	1.42	0.96, 2.09	1.38*	1.11, 1.72	1.55	0.86, 2.81

*Note. iADLS: instrumental Activities of Daily Living. bADLs: basic Activities of Daily Living. MMSE: Mini-Mental Status Examination.*^a^*Single*, separated. *p < 0.05. **p < *0.001*

## Discussion

The results of this study support the hypothesis that trajectories with cognitive decline are associated with higher mortality. We have found a cognitive trajectory gradient for mortality by which individuals with a moderate-stable cognitive trajectory (Class 2) and those in the low-and-declining cognitive function over time (class 3) had a 24% and 96%, respectively, higher risk of death compared to those with the best cognitive trajectory (Class 1-high-to-moderate). We have also found support for the second hypothesis: predictors of mortality were different for each cognitive trajectory. While age and sex were associated in all the classes, showing statistically significant lower risks of death for those younger and women, hypertension increased the risk of death only for those in the high-to-moderate cognitive trajectory; being single (compared to those with a couple), having diabetes, being dependent on basic ADLs, exdrinker, and smoker and/or ex-smoker increased the risk of death only for those in the moderate-stable Class 2; and anxiety almost tripled the risk for individuals in the low-and-declining class 3.

Prior research on the impact of cognitive trajectories, identified by growth mixture and group-based trajectory models, on mortality risk is scarce. Four studies conducted in other cultures used similar cognition assessments and analogous procedures for modeling the trajectories, although the follow-up was shorter in all of them [16, 20, 21, 31]. In line with our results, they showed that the risk of death was higher in the groups who declined more and/or faster, than in the more stable groups. However, only the study of Zang et al. showed a gradient of death risk as we have illustrated here [31]. Wu et al. found a linear association for four of the five trajectories identified [16], and Hu et al. reported that the mortality in the four categories they identified followed a similar hierarchy of the cognitive aging trajectories [20]. The Taniguchi et al. study identified five trajectory groups and found a higher all-cause mortality risk for the lowest group [21]. The smaller size and the shorter follow-up in this last study could to some extent explain the divergence of results. Similarly to our findings, all these studies account for the heterogeneity in the aging process as there were different mortality risks for the groups of trajectories they illustrated.

The association of cognitive decline through aging and mortality has also been reported in studies using different methodologies. Yaffe et al. classified participants based on thresholds of cognitive change, but our modeling of cognitive aging trajectories allowed us to group participants by intra and inter-variability of change, which captures better the natural heterogeneity in the aging process [19, 32]. In a recent and relevant study in the USA, Walsh et al. documented that the rate of change in cognitive trajectories, especially a more rapid linear decline, predicted the risk of mortality above and beyond the initial cognitive level [15]. However, their sample size was much smaller than ours, and the aim of the study was different, intended to analyze to what extent individuals vary in the age profiles of cognitive change and associated incidence risk of dementia, accounting for the competing risk of non-dementia mortality. Moreover, the methods in the Walsh et al. study were also different and simultaneously modeled longitudinal cognitive change and time-to-dementia/death processes.

To our knowledge, this is the first study to test and document differences in the probability of mortality between the highest-and-more-stable and the high-medium/moderate-stable groups, emphasizing the relevance of identifying not only the more pathological trajectories but also differences between the healthier groups, because this can guide more personalized interventions and preventive strategies.

Besides the cognitive trajectories, we have displayed other factors that influenced the risk of death, such as age, masculine sex, civil status, hypertension, diabetes, anxiety, functional dependence, alcohol intake, or smoking status. Education was not associated with mortality in the adjusted model. Nevertheless, the proportions of illiterate individuals among the deceased was significantly higher than in survivors. Prior general studies in the literature have consistently reported an inverse association between education and mortality [33, 34]. Our finding might be explained by the fact that education was codified as a categorical variable, however, a separate sensitivity analysis with a continuous variable (not shown here) showed similar results. Although the lack of association was observed only when the cognitive trajectories classification variable was included the model, the statistical parameters (FIV and tolerances) did not confirm collinearity between education and the trajectories variable. Therefore, a potential statistical explanation is the “mediation” effect, which means that the trajectories might be capturing part of the effect of education on mortality. Furthermore, the scant available data on education as a covariate in comparable previous reports on cognitive trajectories and mortality is consistent with ours. The study of Zang et al. showed that education was not associated with the risk of death in the multivariate-adjusted analysis [31], and Wu et al. detected that subjects in the lower-functioning trajectories had fewer years of education but education did not change their association with increased mortality risk [16].

The present study is also the first to document the predictors of mortality for each cognitive trajectory. To note that the trajectory pattern of the moderate-stable cognitive class, with medium risk of mortality, is the most prevalent in our community sample of subjects older than 55, regardless of their outcome at follow-up; and variables such as being single compared with having a couple, diabetes, dependency on basic ADLs and unhealthy lifestyles were only associated with increased mortality risk in subjects that followed this cognitive trajectory.

While the global analysis in this study, consistently with previous research, documented significant associations between key health variables such as hypertension, diabetes, dependency in ADLs, and alcohol or tobacco use (Table 2), the significance was lost in several variables in the analysis of the trajectories, the loss being remarkable in category 3, the one with most severe cognitive decline. The variable hypertension may be particularly important, since it affects more than 30% of adults worldwide, and is even more prevalent among older individuals [35]. In the stratified analyses we found the association of hypertension and mortality only for those in the highest cognitive trajectory class 1. Hypertension is a known major risk factor for cardiovascular disease mortality and has been associated with a higher systemic immune-inflammation index and suggested as a predictive tool to identify those at risk for chronic diseases [36]. However, the relationship of blood pressure and hypertension with cognition, dementia, and mortality seems complex. Duan J et al. analyzed the potential modifier effect of blood pressure and hypertension on the association between cognition and all-cause mortality in a sample of oldest old individuals and found that both, high blood pressure, as well as hypertension, increased mortality risk in those groups of octogenarians and nonagenarians who suffered cognitive decline. Besides the different methodology, since they did not modelize the trajectories and just used differences on MMSE scores, the effect of hypertension was not described in all the groups of cognitive maintainers which is also intriguing [37]. Recently, we have reported a protective effect on cognitive prognosis for individuals older than 75 years with higher systolic blood pressure levels; and the mortality risk showed a U-shaped association, with the lowest risk at 134 mmHg in subjects aged 60 to 70 and at 155–166 mmHg for age groups between 70 and 95 [38]. Analogous studies analyzing the effect of hypertension on mortality for different cognitive trajectories are needed to evaluate all these findings.

The lack of significant associations between adverse cognitive profiles (class 3) and key health variables prompted further analysis to explore the potential role of incident dementia. During follow-up, 69 individuals in class 3 (29.1%) developed incident dementia, and their mortality rate (79.7%) was significantly higher than that of non-cases (42.9%) (*p* = 0.000). Sensitivity analyses excluding incident dementia cases yielded similar findings, with the exception that the association between sex and mortality lost statistical significance (data not shown).

We also investigated the associations of anxiety and depression with mortality. Notably, anxiety nearly tripled the risk of mortality in the low-and-declining cognitive trajectory class (class 3), an association stronger than those observed for age or sex. Evidence linking anxiety to mortality in older adults is both limited and inconsistent, with some studies reporting an increased risk [39] and others suggesting a protective effect [40]. Possible mechanisms in cases of death include anxiety as a manifestation of vascular brain changes that may ultimately lead to mortality [41]. In contrast, although the proportion of cases of depression were higher in the cases of mortality than in the non-cases, the differences were non-significant, and no significant association between depression and mortality was identified. The lack of association in the low-and-declining cognitive trajectory was unexpected. Severe cognitive impairment has been associated with mortality and the worse cognitive trajectories have been found to be associated with subsequent depressive symptoms, and mortality risks [31]. However, some discrepant results have also been reported: in a 12-year prospective naturalistic study of mortality in individuals age 50 or older with or without cognitive impairment, the association of depressed mood with mortality did not significantly differ by cognitive group [42]. Moreover, a possible explanation for the negative findings in our study might be related to the characteristics of the sample, since differently from most previous studies we excluded at baseline the cases of dementia, but also all cases with cognitive impairment. Given that depressive symptoms and cognitive function in later life have been considered interrelated disorders [43], cases with cognitive impairment and concomitant depression would be probably excluded.

The mortality risk associated with worsening cognitive trajectories, which we document here and has been reported in previous studies, has public health implications since it has been shown that this association was stronger than with factors such as poor health status or depressive symptoms [31]. The current study contributes important new information to the literature by providing a better understanding of the factors that influence mortality risk according to an individual´s cognitive trajectory. Among other strengths of this study, we consider our large sample and repeated measures data from a representative population sample that allowed us to identify, with GMM methodology, cognitive trajectories during follow-up. The observation of mortality six years after the identification of the cognitive trajectories provides robustness to our results.

There are limitations to be acknowledged including those related to response rate or longitudinal dropout. The present study recruited, with a very stringent procedure, individuals who were cognitively healthy at baseline, therefore impeding the observation of the risk of mortality of trajectories in individuals with dementia or with non-severe cognitive deficits at baseline. Furthermore, while several known risk factors of mortality have been included in the analyses, we cannot discard the influence of factors uncontrolled in this study, such as body mass index, grip strength, or APOE e4.

**In conclusion**, this study supports the hypothesis that trajectories with cognitive decline are associated with higher mortality, the risk of death showing a gradient, with the highest risk in those individuals grouped in the category with the low-and-declining cognitive function over time. We have also documented for the first time in the literature that predictors of mortality differ by cognitive trajectory category, the differences being observed even between the cognitively healthier groups. From a practical perspective, these findings underscore the potential for personalized interventions to mitigate mortality risk. For individuals with the best cognitive function, addressing hypertension warrants attention. In those with moderate and stable cognitive function, managing diabetes, basic ADL impairments, and lifestyle factors such as former alcohol consumption and smoking may be critical. Furthermore, the strong association between anxiety and mortality risk highlights the importance of integrating anxiety management into preventive strategies. Nevertheless, further research is needed to validate these predictive models and evaluate their applicability in diverse populations and settings.

## Data Availability

The data presented in this study are available upon reasonable request to the corresponding author.
